# Estimation of renal function and its potential impact on carboplatin dosing in children with cancer

**DOI:** 10.1038/sj.bjc.6604612

**Published:** 2008-08-19

**Authors:** G Chinnaswamy, M Cole, A V Boddy, M Keir, L Price, A Parry, M English, G J Veal

**Affiliations:** 1 Northern Institute for Cancer Research, University of Newcastle upon Tyne, Newcastle upon Tyne NE2 4HH, UK; 2 Regional Medical Physics Department, Royal Victoria Infirmary, Newcastle upon Tyne NE1 4LP, UK; 3 Department of Child Health, Royal Victoria Infirmary, Newcastle upon Tyne NE1 4LP, UK; 4 Department of Oncology, Birmingham Children's Hospital, Birmingham B4 6NH, UK

**Keywords:** paediatrics, carboplatin, renal function, glomerular filtration rate

## Abstract

Renal function-based carboplatin dosing is used routinely in paediatric oncology clinical practice. It is important that accurate assessments of renal function are carried out consistently across clinical centres, a view supported by recently published British Nuclear Medicine Society (BNMS) guidelines for measuring glomerular filtration rate (GFR). These guidelines recommend the use of a radioisotope method for GFR determination, with between two and five blood samples taken starting 2 h after radioisotope injection and application of the Brochner-Mortensen (BM) correction factor. To study the likely impact of these guidelines, we have investigated current practices of measuring GFR in all 21 Children's Cancer and Leukaemia Group (CCLG) paediatric oncology centres in the United Kingdom. This information was used to evaluate the potential impact on renal function-based carboplatin dosing using raw ^51^Cr-EDTA clearance data from 337 GFR tests carried out in children with cancer. A questionnaire survey revealed that between two and four samples were taken after isotope administration, with BM and Chantler corrections used in 38% (8/21) and 28% (6/21) of centres, respectively. A change from Chantler to BM correction, based on the BNMS guidelines, would result in a >10% decrease in carboplatin dose in at least 15% of patients and a >25% decrease in 2% of patients. A greater proportion of patients would have an alteration in carboplatin dose when centres not using any correction factor implement the BM correction. The increase in estimated ^51^Cr-EDTA half-life observed by omitting the 1 h sample decreases carboplatin dose by >10% in 23–52% of patients and by >25% in 3% of patients. This study highlights current variations in renal function measurement between clinical centres and the potential impact on carboplatin dosing. A standard methodology for estimating GFR should be followed to achieve uniform dosing in children with cancer.

Carboplatin dosing based on renal function has been shown to result in more reproducible and reliable drug exposures than dosing based on surface area in children (Thomas *et al*, 2000). This approach not only minimises the risk of underdosing and hence inadequate treatment, but also reduces overdosing, which may be associated with the risk of increased toxicity. As renal function-based carboplatin dosing is now used routinely in clinical practice in many multicentre protocols, it is important that accurate assessments of renal function in children are carried out consistently across paediatric oncology clinical centres.

Glomerular filtration rate (GFR) estimation is widely used as a standard measure of renal function in both children and adults. There are many techniques described in the literature to estimate GFR, using clearance of either exogenous or endogenous markers (Schwartz *et al*, 1976; Price and Finney, 2000; Wright *et al*, 2001; Leger *et al*, 2002; Cole *et al*, 2004; Brandt *et al*, 2006). Use of exogenous markers for estimating GFR involves administration of the marker as either a bolus injection or an infusion and measurement of concentrations in serial blood and/or urine samples over a period of time. Radioisotopically labelled inert substances have been in widespread use and the most common tracers that have been used are iothalmate (^131^I) (Sigman *et al*, 1966), iodothalmate, chromium ethylenediamine tetracetic acid (^51^Cr-EDTA) and technetium diethylenetriamine pentacetic acid (^99^Tc-DTPA) (Chantler and Barratt, 1972; Hilson *et al*, 1976; Price and Finney, 2000). The radioisotope tracers ^51^Cr-EDTA and ^99^Tc-DTPA have found widespread use in paediatric oncology and they are now accepted as a simple, safe and accurate way of measuring GFR. In the United Kingdom, it is standard practice in the majority of Children's Cancer and Leukaemia Group (CCLG) centres to estimate GFR using ^51^Cr-EDTA, whereas in the United States, ^99^Tc-DTPA is the radioisotope tracer of choice (Fleming *et al*, 2004).

Estimation of GFR from the clearance of radiolabelled tracer is most accurate if multiple blood samples are taken after administration as the decrease in plasma concentration of the tracer is often biexponential, with a rapid early phase and a later slower phase (Piciotto *et al*, 1992). However, for routine clinical practice, sampling is usually restricted to the second phase of the clearance, with the slope–intercept method commonly used for the determination of GFR. As the more rapid early phase of the clearance is not accounted for when using this method, it is necessary to correct for systematic errors introduced into the derived GFR values. The most popular methods used to correct for these errors are known as the Chantler and Brochner-Mortensen (BM) corrections (Brochner-Mortensen, 1972; Chantler and Barratt, 1972). Whereas the former uses a constant multiplicative correction factor to adjust the GFR values obtained, the latter uses a quadratic correction and is dependent on the individual's body surface area.

In 2004, the British Nuclear Medicine Society (BNMS) published guidelines to assist nuclear medicine specialists in performing GFR studies and interpreting and reporting the results obtained (Fleming *et al*, 2004). These guidelines recommended that all centres should take between 2 and 5 samples, starting 2 h after injection of radioisotope, and that the GFR should be calculated using the slope–intercept method with the BM correction factor applied.

As carboplatin dosing represents the main practical outcome of estimating GFR in paediatric oncology, it would seem sensible to consider the potential effect that a change in methodology for estimating GFR may have on carboplatin dosing in children with cancer. Therefore, to study the likely impact of the BNMS recommendations, a questionnaire survey concerning the current practices of estimating GFR in children was sent to all CCLG paediatric oncology centres in the United Kingdom. This information was then used to evaluate the potential impact of these changes on renal function-based carboplatin dosing, using ^51^Cr-EDTA clearance data from over 300 GFR tests carried out in a paediatric patient population.

## Patients and methods

### Questionnaire survey

A survey was conducted by means of a questionnaire circulated to all 21 CCLG paediatric oncology centres in the United Kingdom. The questionnaire was sent to centres in May 2006 and all replies were returned by October 2006. The questionnaire was completed by a paediatric oncology consultant or paediatric oncology research nurse in conjunction with the local medical physicist at each centre. The survey requested detailed information regarding the methodology currently practiced in estimating GFR in paediatric oncology patients. The questionnaire also collected details on the existing practice of estimating carboplatin dose based on patient renal function in children with cancer. A follow-up survey was carried out in February 2008 to establish whether any changes had been made to centre practices since the completion of the original questionnaire.

### Impact of BNMS recommendations on carboplatin dosing

Retrospective data from 337 GFR tests carried out on a total of 178 children in Newcastle upon Tyne were used to study the impact of the new BNMS recommendations on carboplatin dosing. These patients had a median weight of 36.6 kg (range: 5.0–120.6 kg) and a median age of 12.4 years (range: 0.2–27.4 years). The raw data used for this analysis were from tests conducted in children with blood samples taken at 1, 2, 3 and 4 h after injection of ^51^Cr-EDTA and covered a wide range of GFR values. A median GFR value of 64.7 ml min^−1^ (range: 9.7–202.1 ml min^−1^), or 95.9 ml min^−1^ 1.73 m^−2^ (range: 14.7–171.2 ml min^−1^ 1.73 m^−2^), was determined in this patient population, based on the data obtained from blood samples taken at 2, 3 and 4 h and application of the BM correction. This involved fitting the data from the 2, 3 and 4 h samples to a single exponential equation by taking the natural logarithm of the plasma concentrations and use of linear regression analysis against time to determine the slope and intercept at time zero for the exponential. GFR_(SI),_ the clearance obtained from these data ((−1 × dose × slope)/intercept), was then modified, according to the BM equation developed for use in children (Brochner-Mortensen *et al*, 1974), to account for the more rapid early phase of the clearance. To obtain the BM modified estimate of clearance GFR_(BM)_, the following three steps were followed:

(1) the BSA corrected, slope–intercept estimate of GFR is given by: 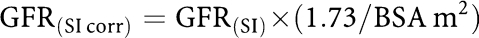


(2) the BSA corrected and BM-modified estimate of GFR is given by: 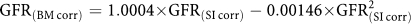


(3) the BM modified estimate of GFR is given by: 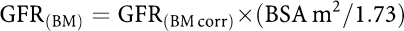


This data set from 337 GFR tests was used to estimate the difference in carboplatin dose that would be seen after implementation of the new regulations in individual centres. Separate analyses were performed to assess the impact of omitting the 1-h blood sample and the implementation of the BM correction in centres where this was not current practice, i.e., in centres either applying the Chantler correction or using no correction factor. The Newell formula based on patient weight and ^51^Cr-EDTA *t*_1/2,_ equation (1), was used to estimate the carboplatin dose to assess the impact of changing sampling times. This involved the determination of ^51^Cr-EDTA *t*_1/2_ using the equation: *t*_1/2_=0.693/elimination rate constant, i.e., using the slope determined from linear regression of the exponential as described above. The alternative Newell formula based on uncorrected GFR and patient weight (modified Calvert formula), equation (2), was used to estimate the carboplatin dose when centres implemented a change in the correction factor used (Newell *et al*, 1993). Both of these formulae were developed for use in a paediatric patient population and the comparisons selected, i.e., the effect of blood sampling on use of the Newell formula and effect of correction factor on use of the modified Calvert formula, were based on the most practical combinations with likely clinical relevance. 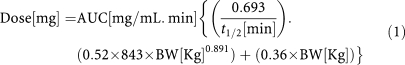




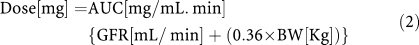



For the purpose of this analysis, a change in carboplatin dose of more than 10% was considered to be of potential clinical relevance.

## Results

### Measurement of GFR

A summary of the findings from the questionnaire relating to current practices in determining renal function in children with cancer at the UK centres is provided in Table 1. The findings indicated that at the time the survey was carried out all centres followed the BNMS guidelines with respect to the use of a radioisotopic method to measure GFR, with 19 of 21 centres using ^51^Cr-EDTA and the remaining two centres using ^99m^Tc-DTPA. Either the central or peripheral route was used to inject the radioisotope tracer in two-thirds of centres, depending on whether the patient had a single or a double-lumen central venous line, whereas the remaining third used the peripheral route only. The BNMS recommendations indicate the use of one lumen of a double-lumen central venous line to inject the radioisotope and the second for blood sampling wherever possible. In situations where only a single-lumen central venous line is present, the peripheral route should be used to inject the radioisotope tracer and the blood samples taken from the central line.

The number and timing of blood samples taken after injection of radioisotopic tracer varied markedly between centres, with six centres taking two blood samples, 10 centres taking three samples and five centres collecting four blood samples. An early blood sample was taken within 2 h of radioisotope administration in 28% (6/21) of centres, potentially falling within the initial rapid exponential phase of disappearance of the radioisotope. Inappropriate inclusion of this early sample in renal function estimation could potentially lead to the inaccurate determination of a reduced ^51^Cr-EDTA *t*_1/2_, an increased GFR and resultant overdosing of carboplatin.

To obtain GFR estimates from the ^51^Cr-EDTA raw data, all but one centre (20/21) applied the slope–intercept method, whereas the remaining centre used an undefined ‘in-house’ method to determine renal function. Among those centres who applied the slope–intercept method, 40% (8/20) used the BM correction, whereas 30% (6/20) applied the Chantler correction. The remaining six centres (30%) applied no correction factor. In the majority of centres (16/21), the Medical Physics department was responsible for estimating GFR from the ^51^Cr-EDTA data obtained, whereas this task was carried out by the Biochemistry department in the remaining five centres.

### Renal function-based carboplatin dosing

All CCLG centres involved in the treatment of children with cancer in the United Kingdom routinely use renal function-based carboplatin dosing as part of chemotherapy regimens for many different tumour types. Estimation of carboplatin dose was carried out solely using ^51^Cr-EDTA *t*_1/2_ values in 52% (11/21) of centres, whereas an additional six centres (29%) used either ^51^Cr-EDTA *t*_1/2_ or uncorrected GFR obtained from the EDTA data. The remaining 10% (2/21) of centres exclusively used uncorrected GFR as the renal parameter, in conjunction with patient body weight, to estimate the carboplatin dose to be administered.

### Glomerular filtration rate measurement and carboplatin dosing

Implementation of the BNMS guidelines, i.e., using data obtained from blood samples taken at 2, 3 and 4 h and application of the BM correction, resulted in a median EDTA *t*_1/2_ value of 93.5 min (range: 47.4–481.8 min) and a median carboplatin dose (calculated to achieve an AUC of 5.0 mg ml^−1^ min^−1^) of 416 mg (range: 72–1207 mg) in this patient population. On the basis of the above findings from the questionnaire survey, implementation of these guidelines would result in the omission of data obtained from early blood samples taken within 2 h of radioisotope administration in six centres. In addition, 12 centres would shift from either applying no correction, or using the Chantler correction, to implement the BM correction. The consequences of these changes on renal function-based carboplatin dosing are summarised in Tables 2 and 3.

**Figure 1:**
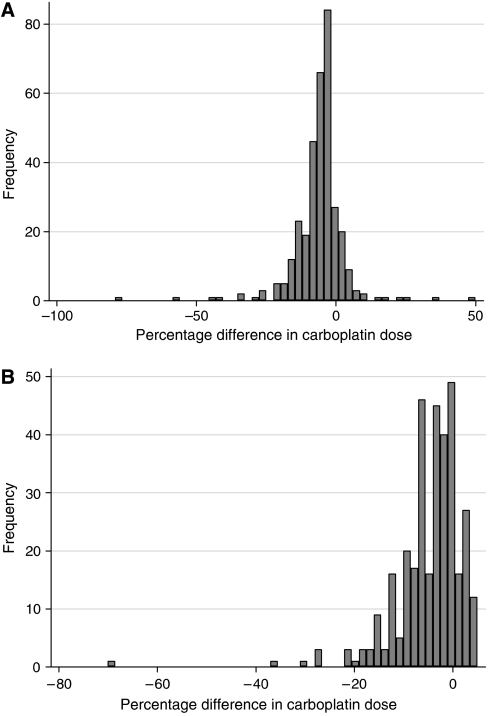
Distribution of percentage change in carboplatin dose following a change in sampling times from 1, 2, 3 and 4 h to 2, 3 and 4 h (**A**), and following a shift from the Chantler correction to the Brochner-Mortensen correction with sampling times at 2, 3 and 4 h (**B**). A decrease in dose is seen for the majority of patients in both cases.

These data show the percentage of children who would have a >10% change in carboplatin dose following the implementation of the BNMS recommendations, including both increases and decreases to the calculated carboplatin dose. Following omission of the early blood sample in those centres where this was taken, the ^51^Cr-EDTA *t*_1/2_ increased in the majority of patients, with a resultant decrease in dose of carboplatin calculated on the basis of ^51^Cr-EDTA *t*_1/2_ and weight as expected. A change in carboplatin dose of >10% would be seen in 23–52% of patients treated at these centres, depending on the existing sampling times in place. The vast majority of patients would have a decrease in the dose of carboplatin administered following the omission of the early blood sample, as illustrated in Figure 1A. In a small group of patients (3%), the dose difference could be as high as 25%.

With regards to the slope–intercept correction method used, a shift from the Chantler correction to the BM correction would result in a >10% change in carboplatin dose in at least 15% of children (Table 3). In a small group of patients (2%), the change in dose could be as high as 25%. In centres applying no correction factor, introduction of the BM correction would impact on a greater proportion of patients, with a >10% change in carboplatin dose observed in 72–85% of children; the change in dose could be as high as 25% in 6–14% of patients. Although the proportion of patients who would experience a significant carboplatin dose change is substantial, the actual mean percentage dose change would be relatively small, ranging from 14–17% across different centres.

Introduction of the BM correction (instead of the Chantler or no correction) would be expected to result in a higher estimate of AUC and the clearance of ^51^Cr-EDTA would decrease. Consequently, this would lead to a decrease in the dose of carboplatin calculated using the Newell formula on the basis of uncorrected GFR and weight. An example of the distribution of carboplatin dose adjustment when the Chantler correction is substituted by the BM correction is illustrated in Figure 1B.

## Discussion

There are numerous techniques described in the literature for estimating renal function or GFR in children and adults (Price and Finney, 2000; Wright *et al*, 2001; Leger *et al*, 2002; Cole *et al*, 2004; Brandt *et al*, 2006). Although creatinine clearance is a reliable marker in day-to-day practice in adults, its use in paediatrics has been questioned and there are situations where a more accurate measure of GFR is preferred (Finney *et al*, 2000; Holweger *et al*, 2005). This information may be particularly important in clinical situations where potentially nephrotoxic drugs are administered, in addition to allowing for accurate renal function-based dosing of anticancer drugs such as carboplatin (Calvert *et al*, 1989; Holweger *et al*, 2005). It has been well established that the use of radioisotope tracers, such as ^51^Cr-EDTA and ^99m^Tc-DTPA, are reliable methods for the accurate estimation of GFR (Rehling *et al*, 1984; Fawdry *et al*, 1985). Techniques of limited sampling restricted to the slow exponential phase of radioisotope clearance provide a reasonable compromise to avoid the complexity of measuring GFR using the biexponential method where multiple blood samples need to be taken at earlier time points. This is particularly important in paediatric practice where blood sampling can be a traumatic experience to a child and where blood volumes need to be minimised. However, this approach necessitates the use of an appropriate correction factor to offset the overestimation of GFR by the slope–intercept technique.

An audit carried out by the BNMS previously highlighted the problems of widespread variation in hospital practices for measuring GFR and guidelines have been published in an attempt to standardise the methodologies used. In view of the BNMS recommendations, it was prudent to investigate the potential effects of changes in methodologies for determining GFR on carboplatin dosing in children with cancer. Therefore, this study aimed to investigate current practices for determining GFR in paediatric oncology centres across the United Kingdom and to use the information obtained to investigate the potential impact of the BNMS recommendations on renal function-based dosing of carboplatin in patients.

Information obtained from all 21 CCLG centres responsible for the treatment of children with cancer in the United Kingdom showed that, to comply with the BNMS recommendations, six centres would be required to stop taking early blood samples (less than 2 h after radioisotope injection) and 12 centres would need to implement the use of the BM slope–intercept correction method. These factors are important when we consider that use of a sample taken 1 h after radioisotope injection, potentially falling within the rapid early phase of elimination, in combination with the slope–intercept method, could lead to inaccurate GFR determinations and potential overdosing of carboplatin. Using raw ^51^Cr-EDTA data from 337 GFR tests carried out in children with cancer, in conjunction with the findings of the questionnaire survey, it was possible to study the potential impact of the new recommendations on carboplatin dosing.

On performing the analyses of various possible combinations of sampling times, it was found that changing from the Chantler to the BM correction factor altered the dose of carboplatin estimated by more than 10% in at least 15% of the children studied. In a small group of patients (2%), the dose difference was as high as 25%. However, the impact was even more marked when changing from no correction to the BM correction, with a >10% change in carboplatin dose observed in 72–85% of children. Omitting the 1-h blood sample after injection of the radioisotope increased the estimate of ^51^Cr-EDTA half-life and consequently decreased the dose of carboplatin estimated using ^51^Cr-EDTA half-life and weight. The alteration in the estimated dose was >10% in 23–52% of the children studied and in 3% of patients, the dose would decrease by >25%.

Whether or not the magnitude of these changes in carboplatin dosage would impact on the ultimate clinical outcome has not been prospectively evaluated and the heterogeneity of the cases in our study population (from whom GFR study data were obtained) would make such an analysis difficult. Also, the 10% change of carboplatin dose used for our analysis represents an arbitrary cutoff point. Although subjective, in view of the narrow therapeutic windows for chemotherapy agents, most clinicians would consider a >10% dose change for any chemotherapeutic drug as being significant. As the predominant shift is towards a decrease in carboplatin dose, implementation of the BNMS recommendations should not raise any concerns regarding potential increases in drug toxicity but an impact on efficacy cannot be ruled out.

These findings highlight the potential problem that patients with similar physical characteristics and renal function could receive different carboplatin doses at different centres on the basis of the methodology used to estimate GFR or ^51^Cr-EDTA half-life. Indeed, it is not unlikely for such an occurrence to be observed in patients being treated on the same clinical protocol. As many paediatric oncology patients in the United Kingdom are recruited to national and international trials, it is logical that the methodology of estimating GFR and carboplatin dose estimation should be standardized, so that the outcome data for these trials are not confounded by this factor. This task could arguably be made more straightforward if the Newell formula was universally used for the determination of renal function-based carboplatin dosing in children, as the ^51^Cr-EDTA half-life on which it is based is only dependent on the radioisotope sampling times and does not depend on the choice of correction factor. Implementation of a change in the type of slope–intercept correction method used for determining GFR, in addition to the changes in sampling times, would impact on the majority of CCLG centres (16 of 21) and there may be some reluctance to implement such changes. Despite the publication of the BNMS recommendations in 2004, there remains substantial variation in practice in paediatric oncology centres across the United Kindom. Indeed, in a follow-up survey carried out as part of this study in February 2008, only three centres reported a change to the previously used method of GFR determination. While one centre had adopted the BNMS recommendations during this time interval, changes in the additional two centres related to the inclusion of a third ^51^Cr-EDTA sampling time point and a shift from the use of ^51^Cr-EDTA to iohexol as the radioisotopic tracer, respectively. There has also been confusion between the use of ‘uncorrected GFR’, the parameter used in the modified Calvert formula for estimating carboplatin dose, and body surface area or weight ‘standardized’ or ‘normalized’ GFR with reports in the literature where serious dosing errors have occurred when these values have been misused (Liem *et al*, 2003). The standardised use of ^51^Cr-EDTA half-life as the renal parameter to estimate carboplatin dose would prevent such problems occurring.

In summary, our findings suggest that steps should be taken to implement the new BNMS guidelines and standardise methodologies of GFR estimation and renal function-based carboplatin dose calculation in all centres. Ideally, we recommend the determination of carboplatin dose in children with cancer on the basis of ^51^Cr-EDTA half-life and weight wherever possible.

## Figures and Tables

**Table 1:** Results of the questionnaire survey showing the methodology of estimation of GFR in the UK CCLG centres

**Centre**	**Radioisotope**	**Route of injection**	**No of samples**	**Early sampling (<2 h)**	**GFR estimation**	**Methodology**	**Correction factor**
1	^51^Cr-EDTA	Peripheral	3	Yes	Med Physics	Slope–intercept	BM
2	^51^Cr-EDTA	Central	2	No	Biochemistry	Slope–intercept	Chantler
3	^51^Cr-EDTA	Central	2	No	Biochemistry	Slope–intercept	Chantler
4	^51^Cr-EDTA	Central	4	No	Med Physics	Slope–intercept	Chantler
5	^51^Cr-EDTA	Peripheral	4	No	Med Physics	Slope–intercept	BM
6	^51^Cr-EDTA	Both	4	Yes	Med Physics	Slope–intercept	BM
7	^51^Cr-EDTA	Both	3	No	Med Physics	Slope–intercept	BM
8	^51^Cr-EDTA	Both	3	No	Med Physics	Slope–intercept	Modification
9	^99m^Tc-DTPA	Both	2	No	Med Physics	Slope–intercept	None
10	^51^Cr-EDTA	Both	3	No	Med Physics	Slope–intercept	Chantler
11	^51^Cr-EDTA	Peripheral	3	No	Med Physics	Slope–intercept	BM
12	^51^Cr-EDTA	Peripheral	2	No	Biochemistry	Slope–intercept	None
13	^51^Cr-EDTA	Both	2	No	Biochemistry	Slope–intercept	Chantler
14	^51^Cr-EDTA	Both	3	Yes	Med Physics	Slope–intercept	None
15	^51^Cr-EDTA	Peripheral	3	No	Med Physics	Slope–intercept	None
16	^51^Cr-EDTA	Both	4	No	Med Physics	Slope–intercept	BM
17	^99m^Tc-DTPA	Both	2	No	Med Physics	Slope–intercept	BM
18	^51^Cr-EDTA	Both	3	Yes	Med Physics	In-house method	None
19	^51^Cr-EDTA	Peripheral	3	Yes	Biochemistry	Slope–intercept	None
20	^51^Cr-EDTA	Central	4	Yes	Med Physics	Slope–intercept	Chantler
21	^51^Cr-EDTA	Peripheral	3	No	Med Physics	Slope–intercept	BM

BM=Brochner-Mortensen; CCLG=Children's Cancer and Leukaemia Group; GFR=glomerular filtration rate.

**Table 2:** Predicted impact on renal function-based carboplatin dosing of a change from existing sampling times to the new BNMS guidelines

**Existing sampling times (h)**	**New sampling times (h)**	**Number of centres**	**Percentage of children who have a >10% change in carboplatin dose**
1, 2, 3 and 4	2,3 and 4	1	23 (21% increase/2% decrease)
1, 2 and 3	2,3 and 4	3	52 (47% increase/5% decrease)
2 and 4	2,3 and 4	5	No change
2, 3 and 4	2,3 and 4	8	No change
1, 3 and 4	2,3 and 4	1	27 (25% increase/2% decrease)
2 and 3	2,3 and 4	1	33 (23% increase/10% decrease)
Others	—	2	—

BNMS=British Nuclear Medicine Society.

Dosing on the basis of ^51^Cr-EDTA *t*_1/2_ and weight.

**Table 3:** Predicted impact on renal function-based carboplatin dosing of a change from current practices to the new sampling times and correction factor recommended by BNMS

**Existing sampling times (h)**	**Existing correction factor**	**New sampling times (h)**	**New correction factor**	**Number of centres**	**Percentage of children with >10% carboplatin dose change**
1, 2, 3 and 4	None	2,3 and 4	BM	1	72
1, 2 and 3	None	2,3 and 4	BM	3	80
2 and 4	None	2,3 and 4	BM	2	85
2, 3 and 4	None	2,3 and 4	BM	1	85
2, 3 and 4	Chantler	2,3 and 4	BM	2	15
2 and 4	Chantler	2,3 and 4	BM	3	15
1, 3 and 4	BM	2,3 and 4	BM	1	4
2 and 3	BM	2,3 and 4	BM	1	4
2, 3 and 4	BM	2,3 and 4	BM	5	No change
Others	—	2,3 and 4	BM	2	Not applicable

BM=Brochner-Mortensen; BNMS=British Nuclear Medicine Society.

Dosing based on uncorrected GFR and weight.
